# Supplementary material for: Early Mobilization of Critically Ill Patients: A Survey of Knowledge, Practices and Perceptions of Greek Physiotherapists
Source: Healthcare (Basel). 2025 May 26;13(11):1248. doi: 10.3390/healthcare13111248 (PMC12154428; doi:10.3390/healthcare13111248)
Supplement: Supplementary file 1 [file healthcare-13-01248-s001.zip › healthcare-3637836-supplementary.pdf]

Supplementary Table S1. Items measuring Greek physiotherapists' knowledge on EM practice

Items	Questions	Responses	
1	Do you know the ESICM guidelines regarding the prevention of post ICU syndrome?	Yes	44%
		No	39%
		I don't know	17%
2	Do you know what the process of early mobilization in the ICU involves (techniques)?	Yes	81%
		No	16%
		I don't know	3%
3	Do you know the benefits of early mobilization in the ICU?	Yes	94%
		No	4%
		I don't know	2%
4	Do you know the criteria for safely initiating early mobilization of patients in the ICU?	Yes	70%
		No	21%
		I don't know	9%
5	Do you know the criteria for discontinuing the mobilization of patients in the ICU?	Yes	75%
		No	17%
		I don't know	8%
6	Do you know the contraindications of implementing early mobilization in the ICU?	Yes	78%
		No	14%
		I don't know	8%

Supplementary Table S2. Items that measure practices related to EM in Greek ICUs

Items	Questions	Responses		
		Yes	Don't Know	No
1.	Do you use the ABCDEF bundle in your ICU?	31%	44%	25%
2.	Do physiotherapists participate in the morning briefing of physicians?	32%	1%	67%
3	Do you require a physician's order before early mobilization?	86%	1%	13%
4	Is there a time delay in initiating EM?	40%	22%	37%

5	Do you record the daily progress of the patient's mobilization plan?	40%	3%	57%
6	Do you follow a protocol regarding the process of early mobilization of patients in the ICU?	23%	6%	71%
7	Do you follow clinical guidelines regarding EM?	55%	4%	41%

Supplementary Table S3. Items that measure perceptions of Greek physiotherapists regarding EM

Items	Statements			
		Agree	Neutral	Disagree
1.	The implementation of early mobilization of patients in the ICU is important for preventing complications of immobility.	99%	1%	0%
2.	Early mobilization of patients in the ICU is a priority for their rehabilitation.	85%	12%	3%
3.	The implementation of early mobilization of patients in ICU is safe.	77%	5%	8%
4.	The risks of early mobilization are more than its benefits.	22%	14%	64%
5.	Intensivists are in favor of early mobilization.	73%	20%	7%
6.	The ICU nurses are in favor of early mobilization.	30%	43%	27%
7.	Conducting specialized seminars/ training sessions will contribute to improving the implementation of early mobilization in the ICU.	96%	3%	1%
8.	Ergonomic training is important for the safe implementation of early mobilization techniques.	97%	3%	0%

9.	Staying informed about professional developments in ICU physiotherapy in the international scientific community is important.	96%	4%	0%
10.	Having a protocol for early mobilization of ICU patients is important for its safe implementation.	92%	8%	0%
11.	Creating a bundle for early mobilization of ICU patients is important for its safe implementation.	96%	4%	0%

Supplementary Table S4. Items that measure perceived barriers to EM

Items		Responses		
	Patients' barriers to early mobilization	Agree	Neutral	Disagree
1.	Obesity	26%	1%3	61%
2.	Pain	42%	29%	29%
3.	Hemodynamic instability	94%	3%	3%
4.	Incoherence with the ventilator	61%	25%	14%
5.	Muscle weakness	11%	10%	79%
6.	Severity of illness	33%	29%	37%
7.	Poor nutritional status	18%	26%	56%
8.	Endotracheal tube	6%	13%	81%
9.	Femoral line	8%	10%	82%
10.	Hemodialysis session	36%	34%	30%
11.	Delirium	48%	28%	24%
12.	Excessive sedation	49%	15%	36%
13.	Fatigue	34%	33%	33%
14.	Palliative care	15%	14%	71%
15.	Patient's refusal	41%	28%	31%
	Institutional and providers' barriers to early mobilization			
1.	Adequate equipment	36%	24%	40%

2.	Adequate shift time regarding number of patients	49%	18%	33%
3.	Adequate number of physiotherapists	42%	13%	45%
4.	ICU training	98%	2%	0%
5.	Training to facilitate early mobilization	94%	6%	0%
6.	The existence of protocol regarding the process of early mobilization of patients in the ICU is essential	92%	6%	0%
7.	The existence of clinical guidelines regarding the process of early mobilization of patients in the ICU is essential.	96%	3%	1%
	Barriers related to the process of early mobilization			
1.	Physician's order required before early mobilization .	86%	1%	13%
2.	Delayed decision on appropriate time of Initiation.	40%	22%	37%
3.	Conflicting perceptions regarding the safe implementation of early mobilization.	67%	13%	20%
4.	Lack of Coordination during early mobilization with other ICU staff .	62%	2%	36%
5.	Patient's early mobilization out of the ICU.	44%	15%	41%
6.	Lack of communication, coordination, and organization of the therapeutic interventions that need to be carried out for each patient among the ICU staff.	46%	14%	40%
7.	Musculoskeletal self- injury during early mobilization.	76%	5%	19%
